# Experimental and Theoretical Approaches to Describing Interactions in Natural Cell Membranes Occurring as a Result of Fatal Alcohol Poisoning

**DOI:** 10.3390/membranes11030189

**Published:** 2021-03-09

**Authors:** Aneta D. Petelska, Michał Szeremeta, Joanna Kotyńska, Anna Niemcunowicz-Janica

**Affiliations:** 1 Faculty of Chemistry, University of Bialystok, Ciolkowskiego 1K, 15-245 Bialystok, Poland; joannak@uwb.edu.pl; 2 Department of Forensic Medicine, Medical University of Bialystok, Waszyngtona St. 13, 15-230 Bialystok, Poland; michalszeremeta@gmail.com (M.S.); anna.janica@umb.edu.pl (A.N.-J.)

**Keywords:** fatal ethyl alcohol poisoning, surface charge density, microelectrophoresis, acid–base equilibria, erythrocytes, thrombocytes

## Abstract

We propose herein a theoretical model describing the effect of fatal ethanol poisoning on the equilibria between cell membranes and the surrounding ions. Using this model, we determined the parameters characterizing the interaction between the electrolyte solution’s ions and the functional groups on the blood cells’ surface. Via the application of mathematical equations, we calculated the total surface concentrations of the acidic and basic groups, *c_A_* and *c_B_*, and their association constants with solution ions, *K_AH_* and *K_BOH_*. Using the determined parameters and mathematical equations’ values, we calculated the theoretical surface charge density values. We verified the proposed model by comparing these values with experimental data, which were selected based on measurements of the electrophoretic mobility of erythrocyte and thrombocyte membranes. Compatibility of the experimental and theoretical surface charge density values was observed in the range of pH 2–8, while deviations were observed at higher pH values.

## 1. Introduction

Alcoholism is a severe health problem worldwide. Even if it is not itself the direct cause of death, ethanol abuse is associated with many health risks, including poisoning and the greater spread of HIV and tuberculosis. Alcohol negatively affects various types of blood cells and their functions. Ethyl alcohol abusers most often have defective red blood cells; they are destroyed prematurely, leading to anemia. Drinking too much ethyl alcohol can increase stroke risk as it affects the blood coagulation system [1]. Ethyl alcohol is toxic to the bone marrow (it contains blood cell precursors) and all cells circulating in the blood [2]. Microscopic examination of cultured blood cell precursors has shown that exposure of cells to a wide range of ethyl alcohol concentrations causes damage to the membrane surrounding each cell.

Alcoholism can also modify blood clotting or coagulation. Alcohol consumption can interfere with these processes at several levels, causing thrombocytopenia (abnormally low blood platelet count), thrombocytopathy (impaired platelet function), and reduced fibrinolysis. Researchers have suggested that alcohol intoxication itself causes a decrease in blood platelets. Literature data also indicate that alcohol shortens existing thrombocytes’ lifespan and may affect the late stage of platelet production. Alcohol abuse also affects the properties of thrombocytes. These platelet abnormalities include impaired platelet aggregation and decreased secretion or activity of platelet-derived proteins involved in blood coagulation [3,4,5,6,7].

Even small changes in the composition of cell membranes can strongly affect their functioning and physicochemical properties. These changes may occur due to the modification of cell membranes by short-chain alcohols like ethanol, a particularly important representative of molecules that can modulate membranes’ properties. As a first approximation to understanding the interaction of ethanol with cell membranes, lipid bilayers are considered. This interaction is of biophysical interest as ethyl alcohol can induce the formation of interdigitated bilayer structures and modulate lipids’ phase stability. Numerous experimental and computational studies have been performed to determine how ethyl alcohol affects lipid bilayers [8,9,10]. Although the alcohol has an amphiphilic nature, its hydrophobicity is limited; it can pass through the bilayer. Besides this, ethanol molecules condense near the interface region between lipids and water. –OH groups are positioned in the bilayer interfacial region (forming hydrogen bonds with hydrophilic lipid headgroups), and the hydrocarbon chains face the hydrophobic core of the bilayer. The presence of ethanol in the lipid membrane has a disordering effect on the hydrocarbon chains, giving rise to an increase in the membrane’s area per lipid and fluidity.

The interaction of ethyl alcohol with lipid membranes, both natural and model, has been examined for years. Alcohol-induced modifications in erythrocyte membranes have been extensively investigated using different experimental methods such as Electron paramagnetic resonance spectroscopy (EPR) spectroscopy [11,12], fluorescence anisotropy [13,14], gas chromatography [15], and microelectrophoresis [16,17,18]. Nevertheless, there is limited data concerning the influence of ethanol poisoning on thrombocyte membranes. In the literature, one can find microelectrohoretic studies of cell membranes’ electric properties when intoxicated with ethanol, but it should be emphasized that these reports concern rats. The authors found that alcohol significantly enhanced changes in the surface charge density of erythrocyte membranes [17] and liver cell membranes [18]. The obtained results indicate that changes in cell membrane charge connect with changes in membrane composition.

In recent years, studies of the electrical properties of cell membranes have focused mainly on model membranes—monolayers [19], bilayers [20], and supported lipid membranes [21]—while there are few papers concerning natural membranes [22,23]. The results included in this paper are a continuation of the systematic research on the electrical properties of blood cell membranes conducted by Petelska et al. [24,25,26,27,28]. In this work, we analyze the influence of fatal ethanol poisoning on human blood cell membranes’ surface charge, which is an important parameter describing equilibria in both model and natural membranes.

Erythrocytes and thrombocytes have relatively simple structures. Thus, they are ideal cellular models to study the alterations of membranes’ physicochemical properties under the influence of small amphiphilic solutes, like ethanol. In cellular systems, the toxicity of ethyl alcohol has been suggested to be due to its interactions with membranes [29]. Due to the lack of literature data on the effect of fatal poisoning with ethyl alcohol on the equilibria between the membranes of erythrocytes and thrombocytes and the surrounding environment, we proposed a mathematical model to describe these equilibria. The obtained data indicate the influence of alcohol intoxication on the blood cell membranes. In our opinion, our quantitative description of the properties of erythrocyte and thrombocyte membranes may help to better understand the impact of fatal ethyl alcohol poisoning on the surface properties of blood cell membranes.

## 2. Materials and Methods

### 2.1. Materials

The Ethics Review Board of the Medical University of Bialystok approved the conducted research (No. R-I-002/533/2010). During autopsies made at the Department of Forensic Medicine, Medical University of Bialystok, blood (pH ~ 6.9) was obtained from individuals who died due to fatal alcohol poisoning. The conducted experiment was based on ten selective fatal alcohol poisonings (5 men and 5 women; mean age 45.8 years, range 19–65). Blood was taken for tests from the femoral vein and placed in sterile containers, then sent to the Faculty of Chemistry, University of Bialystok for further research. The obtained results were subjected to a comparative analysis with a control group obtained from live individuals at the Blood Donation Center in Białystok.

RBC isolation: The RBCs were isolated from 2 mL of whole blood by centrifugation for 8 min at room temperature at 900× *g*. The thrombocyte-rich plasma supernatant was removed, and the obtained erythrocytes were washed three times with 0.9% NaCl, then centrifuged for 15 min at 3000× *g*. After the final centrifugation, erythrocytes were resuspended in 0.9% NaCl, followed by microelectrophoretic measurement.

Platelet isolation: The thrombocyte-rich plasma was centrifuged for 8 min at 4000× *g*. The plasma supernatant was removed and discarded. Thrombocytes were washed three times with physiological saline solution (0.9% NaCl) and then centrifuged for 15 min at 3000× *g*. Then, thrombocytes were resuspended in 0.9% NaCl, and microelectrophoretic measurements were made.

The test solutions were made and all purification procedures were performed using ultrapure water from a Milli-Q11 system (18.2 MΩ cm, Millipore, Burlington, MA, USA).

### 2.2. Methods

#### Microelectrophoretic Mobility Measurements

The purpose of the microelectrophoretic experiment was to obtain the dependence of the surface charge density on the pH of the electrolyte solution. Using a Zetasizer Nano ZS (Malvern Instruments, Malvern, UK) apparatus allowed us to obtain data on the microelectrophoretic mobility of the blood cells using the laser Doppler microelectrophoresis (LDE) technique. WTW InoLab 720 laboratory pH meter (WTW, Weinheim, Germany) use for all measurements was performed as a function of pH. Six measurements were made (each covering 100–200 series for a duration of 5 s) for each pH value for each sample.

Equation (1) allowed us to calculate of the surface charge density from electrophoretic mobility measurements [30]:

(1) $$\delta =\frac{\eta \cdot u}{d}$$

where *η* is the viscosity of the solution, *u* is the electrophoretic mobility, and *d* is the diffusion layer thickness.

The thickness of the diffusion layer can be determined from the following formula (Equation (2)) [31]:

(2) $$d=\sqrt{\frac{\varepsilon \cdot \varepsilon_{0}\cdot R\cdot T}{2\cdot F^{2}\cdot I}}$$

where *R* is the the gas constant, *T* is the temperature, *F* is the Faraday number, *I* is the ionic strength of 0.9% NaCl, and *εε*_0_ is the permeability of the electric medium.

## 3. Results

**Figure 1:**
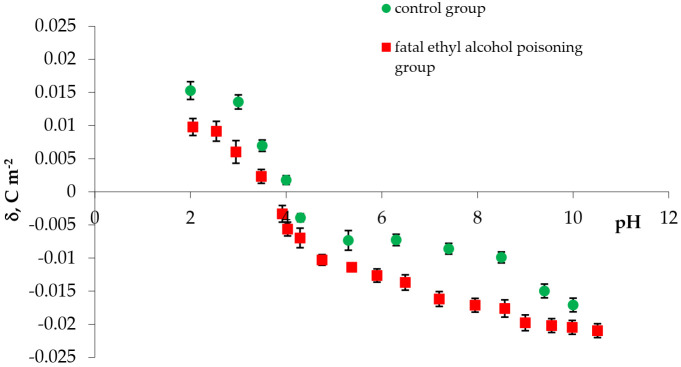
The experimental surface charge density of erythrocytes vs. pH of the electrolyte solution ●—control, ■—fatal ethyl alcohol poisoning.

**Figure 2:**
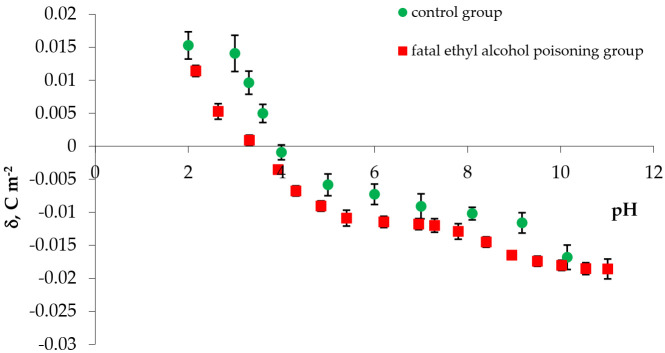
The experimental surface charge density of thrombocytes vs. pH of the electrolyte solution: ●—control, ■—fatal ethyl alcohol poisoning.

The experimental values of the surface charge densities of erythrocyte and thrombocyte membranes (control and fatal ethyl alcohol poisoning groups) versus pH are presented in Figure 1 and Figure 2, respectively. We determined the surface charge density values based on experimental electrophoretic mobility data using Equation (1). All measurements were carried out at several pH values, using the basic electrolyte (0.155 M NaCl).

Figure 1 shows the surface charge densities of the control and fatal ethyl alcohol poisoning erythrocyte groups versus pH values. We observed a decrease in the positive charge and an increase in the erythrocytes membrane’s negative charge after death by ethyl alcohol poisoning compared to control erythrocytes. A slight shift of the isoelectric point of membranes to lower pH values is visible.

Figure 2 shows the effect of pH on the surface charge density for the control and fatal ethyl alcohol poisoning thrombocyte groups.

Fatal ethyl alcohol poisoning caused a decrease in the positive membrane charge and increased the thrombocyte group’s negative charge compared with the control. Also, we observed a shift of the isoelectric point of the membrane to lower pH values.

Table 1 and Table 2 show the isoelectric point values and the surface charge densities of the erythrocyte and thrombocyte membranes, as determined by microelectrophoresis. We express the summarized results as the mean value with the designated standard deviation. We performed the analysis using standard statistical analysis.

## 4. Discussion

The natural cell lipid bilayer is a complex and dynamic protein-lipid structure. The complexity of biological membranes determines many equilibria, both between the membrane components and between cell membranes and the surrounding ions. Particularly important to the cell’s functioning are the equilibria between the membrane and the surrounding aqueous solution. The ions present in the solution, adsorbing on the membrane, modulate many of its physicochemical and electrical properties, such as electric charge. Due to the lack of literature data on the effect of fatal poisoning with ethyl alcohol on the equilibria between blood cell membranes and their surroundings, we adopted the theoretical model proposed by Dobrzyńska et al. [32] (presented in full detail in [24]) to describe these equilibria. The given model allowed us to verify the theoretical data with experimental data.

Equations (1)–(7) describe the assumptions of the presented model. H^+^, OH^−^, Na^+^, and Cl^−^ ions from the electrolyte solution adsorb at the erythrocytes’ and thrombocytes’ surface. Two of the four equilibria described are associated with positive groups (phospholipids or proteins and sodium or hydrogen ions). The other two are associated with negative groups on the phospholipids’ or proteins’ surface and hydroxide or chloride ions. The equations of adsorption of H^+^, OH^−^, Na^+^, and Cl^−^ ions on functional groups located on the membrane surface are presented in Equations (1)–(4):

A^−^ + H^+^ ⇔ AH (3)

A^−^ + Na^+^ ⇔ ANa (4)

B^+^ + OH^−^ ⇔ BOH (5)

B^+^ + Cl^−^ ⇔ BCl (6)

(7) $$c_{A}=a_{A^{-}}+a_{AH}+a_{ANa}$$

(8) $$c_{B}=a_{B^{+}}+a_{BOH}+a_{BCl}$$

(9) $$\delta =\;\left(a_{B^{+}}-a_{A^{-}}\right)\cdot F$$

where $\;a_{AH}$, $a_{ANa}$, $a_{A^{-}}$, $a_{BOH}$, $a_{BCl}$, and $a_{B^{+}}$ are the surface concentrations of the corresponding groups on the membrane surface; $a_{H^{+}}$, $a_{Na^{+}}$, $a_{OH^{-}}$, and $a_{Cl^{-}}$ are the volume concentrations of solution ions; $c_{A}$ is the total surface concentration of the membrane acidic groups; $c_{B}$ is the total surface concentration of the membrane basic groups, F=96,487 $\left[\frac{C}{mol}\right]$ is the Faraday constant; and δ is the surface charge density.

Final equations [24,32]:

- The equation for determining the membrane surface charge density:

(10) $$\frac{\delta }{F}=\frac{C_{B}}{1+K_{BOH}a_{OH^{-}}+K_{BCl}a_{Cl^{-}}}-\frac{C_{A}}{1+K_{AH}a_{H^{+}}+K_{ANa}a_{Na^{+}}}$$

- Linear equations—simplifications of Equation (8)—for high (Equation (9)) and low (Equation (10)) concentrations of hydrogen ions:

(11) $$\frac{\delta a_{H^{+}}}{F}=\frac{C_{B}}{1+K_{BCl}a_{Cl^{-}}}a_{H^{+}}-\left(\frac{C_{B}K_{BOH}K_{W}}{{\left(1+K_{BCl}a_{Cl^{-}}\right)}^{2}}+\frac{C_{A}}{K_{AH}}\right)$$

(12) $$\frac{\delta }{Fa_{H^{+}}}=-\left(\frac{C_{A}}{1+K_{ANa}a_{Na^{+}}}\right)\frac{1}{a_{H^{+}}}+\left(\frac{C_{B}}{K_{BOH}K_{W}}+\frac{C_{A}K_{AH}}{{\left(1+K_{ANa}a_{Na^{+}}\right)}^{2}}\right)$$

where $K_{AH}$, $K_{ANa}$, $K_{BOH}$, and $K_{BCl}$ are association constants.

The above linear function coefficients can be easily determined using the linear regression method and then used to calculate the membrane parameters (Equations (9) and (10)). The necessary parameters are the total concentrations of functional acidic ($c_{A}$) and basic ($c_{B}$) groups on the blood cell surfaces and their average association constants with hydrogen ($K_{AH}$) and hydroxyl ($K_{BOH}$) ions. Determination of all the necessary parameters was possible only based on the assumption that the association constant values $K_{ANa}$ and $K_{BCl}$ are the same as the values obtained for phosphatidylcholine liposomes. The $K_{ANa}$ and $K_{BCl}$ values of the surface groups of phosphatidylcholine with sodium and chloride ions were reported previously and amount to 0.230 and 0.076 [m^3^/mol], respectively [18]. To calculate the theoretical values of the erythrocyte and thrombocyte membranes’ surface charge density, the values of $c_{A}$, $c_{B}$, $K_{AH}$, and $K_{BOH}$ were determined and substituted into Equation (8).

**Figure 3:**
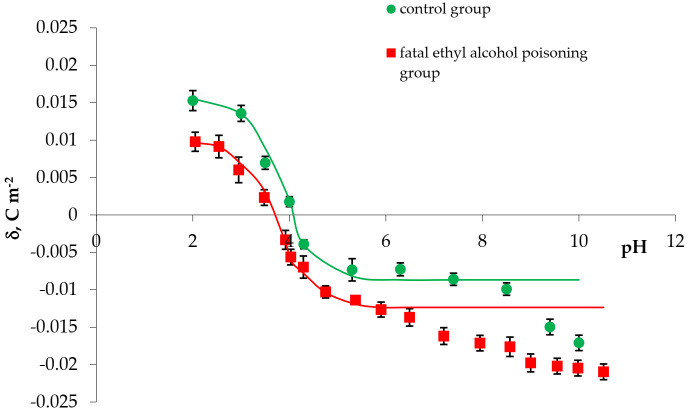
A comparison of experimental and theoretical surface charge density values of erythrocytes vs. pH of the electrolyte solution: ● control, ■ fatal ethyl alcohol poisoning. Points represent the experimental values and curves represent theoretical values.

**Figure 4:**
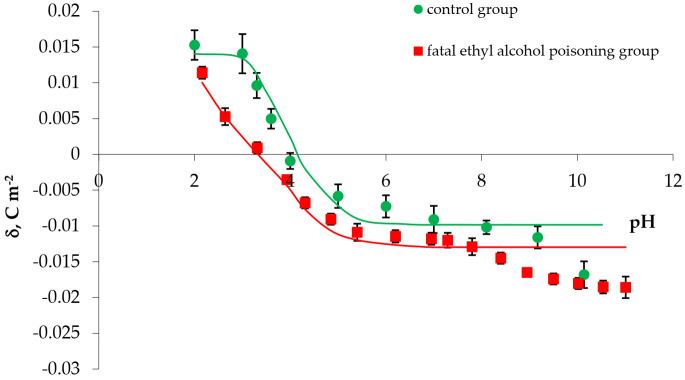
A comparison of experimental and theoretical surface charge density values of thrombocytes vs. pH of the electrolyte solution: ● control, ■ fatal ethyl alcohol poisoning. Points represent the experimental values and curves represent theoretical values.

The comparison of the experimental (calculated from Equation (11)) and theoretical (calculated from Equation (8)) surface charge density values of the erythrocyte and thrombocyte membranes versus pH are presented in Figure 3 and Figure 4 (the points indicate the experimental data and the curves indicate theoretical data).

The following conclusion can be drawn from the curves presented in Figure 3 and Figure 4: There is an agreement between the theoretical and experimental values in the pH range of 2–7. Above pH 7, the theoretical curves differ from the experimental points. Variations at pH above 7 may be caused by interactions between the functional groups of the cell membranes of blood morphotic elements. The mathematical model we proposed takes into account only the equilibrium of the membrane surface with electrolyte ions.

In Table 3 and Table 4, we summarize the obtained values of the parameters characterizing the equilibria between the erythrocyte and thrombocyte surfaces. The presented results were analyzed using standard statistical analysis and are expressed as means with standard deviations. The effect of fatal alcohol poisoning on erythrocyte and thrombocyte membranes caused changes in the values of the calculated parameters ($c_{A}$, $c_{B}$, $K_{AH}$, and $K_{BOH}$) (Table 3 and Table 4).

In this study, the *c_A_*, *K_AH_*, and *K_BOH_* values for an erythrocyte cell membrane were affected by ethyl alcohol and were smaller than the same parameters assayed in unmodified cells; only *c_B_* values were higher (approximately double, Table 3).

In the case of a thrombocyte cell membrane, the *c_A_*, *K_AH_*, and *K_BOH_* parameters were affected by ethyl alcohol and were higher than the same parameters assayed in unmodified cells. Only *c_B_* values were smaller, by about seven times (Table 4).

Alcohol intoxication causes changes in the amounts of both phospholipids and integral proteins of erythrocyte membranes [17], resulting in the appearance of new negatively and positively charged functional groups. Therefore, variations in the kind and number of these groups result in changes in the parameters describing equilibria in cell membranes: *c_A_*, *c_B_*, *K_AH_*, and *K_BOH_*. It is believed that other mechanisms can also influence the above parameters, such as the movement of molecules forming membranes between monolayers (the flip-flop phenomenon) or the appearance of receptor proteins or their components on the surface of cell membranes, which, as a result of stopped life processes, no longer perceive signals from the external environment and remain in an inactive form. Scheidt and Huster [33] demonstrated that ethyl alcohol partitions the lipid–water interface of the phospholipid bilayer by forming hydrogen bonds with lipid molecules and, also, by the hydrophobic effect. Rowe showed that at a higher ethanol concentration, a significant reduction (up to 30%) in phosphatidylcholine lipid membrane thickness is observed as it transitions into the interdigitated phase [34]. In natural lipid membranes, ethanol-induced membrane perturbations may have many possible effects. A considerable reduction in membrane thickness caused by lipid interdigitation would likely profoundly affect membrane protein function and conformation [35]. Membrane thickness changes can result in the exposure of hydrophobic amino acid residuum in integral proteins of the membrane and an occurrence like a hydrophobic mismatch, leading to membrane protein aggregation [36] and possibly producing a conformational change in the membrane protein [37]. Zeng et al. [38] demonstrated that ethanol-induced fluidization and interdigitation of the lipid bilayer lead to an increase in the membrane’s ion permeability. Lee [39] examined the effects of ethanol exposure on human erythrocytes using quantitative phase imaging techniques at the level of individual cells. The author demonstrated that erythrocytes exposed to ethanol (at concentrations of 0.1 and 0.3% *v*/*v*) exhibited cell sphericities higher than those of normal cells. Bulle and co-workers [40] studied the association between erythrocyte membrane alterations and hemolysis in chronic alcoholics. They showed that frequent alcohol consumption increases oxidative/nitrosative stress. The result is a change in the lipid composition of the erythrocyte membrane and the protein content, resulting from increased hemolysis.

There are no data on ethyl alcohol’s effect on blood platelets’ electrical properties in the literature. Slowed blood flow in ethyl alcohol poisoning promotes platelet activation, leading to changes in the exposure of phospholipids (phosphatidylserine and phosphatidylethanolamine) in the clotting process to the surface of the cell membrane. Since phospholipids are endowed with an electric charge, we believe that their presence in the membrane will result in changes in the surface charge density and, consequently, the determined parameters (*c_A_*, *c_B_*, *K_AH_*, and *K_BOH_*).

## 5. Conclusions

Based on numerous literature reports, it can be said that ethyl alcohol shows biophysical effects on lipid membranes. In this paper, both experimental and theoretical data demonstrated that the electrical properties of blood cell membranes (erythrocytes and thrombocytes) are changed upon fatal alcohol poisoning. We observed changes in all determined parameters, $c_{A}$, $c_{B}$, $K_{AH}$, and $K_{BOH}$. Interactions among membrane components (lipids and proteins) and between these components and their surroundings lead to variations in the number and kind of functional groups present, which results in changes in $c_{A}$ and $c_{B}$ and in the values of association constants. Also, sialic acid, a component of glycoproteins and glycolipids, may influence all these parameters in fatal ethyl alcohol poisoning. Understanding some of the physical and chemical processes occurring in the human body after death is essential in describing the pathophysiology of fatal ethyl alcohol poisoning deaths. A quantitative description of the phenomena occurring on the blood cell membrane surfaces and the obtained parameters characterizing the existing equilibria (*c_A_*, *c_B_*, *K_AH_*, and *K_BOH_*) may provide essential information for forensic medicine. We want to emphasize that our theoretical descriptions are innovative and the research carried out is quite preliminary. Still, because the obtained data may help solve many forensic medicine problems, there is no doubt that there is a necessity for continuation of this research.

## Figures and Tables

**Table 1:** The isoelectric point and surface charge density values for red blood cells (RBCs) (control and fatal ethyl alcohol poisoning).

Examined Groups	Isoelectric Point	Surface Charge Density [10^−2^ C m^−2^]	
		pH ~ 3	pH ~ 10
control	4.05	1.357 ± 0.113	−1.710 ± 0.109
fatal ethyl alcohol poisoning	3.92	0.978 ± 0.068	−2.300 ± 0.106

**Table 2:** The surface charge density and isoelectric point values for thrombocytes (control and fatal ethyl alcohol poisoning).

Examined Groups	Isoelectric Point	Surface Charge Density [10^−2^ C m^−2^]	
		pH ~ 3	pH ~ 10
control	4.20	1.407 ± 0.212	−1.681 ± 0.178
fatal ethyl alcohol poisoning	3.31	1.141 ± 0.138	−1.858 ± 0.155

**Table 3:** The acidic and basic total concentrations of functional groups of erythrocytes and their association constants with H^+^ and OH^−^ ions.

Groups	Parameters			
	*c_A_* [10^−6^ mol/m^2^]	*c_B_* [10^−6^ mol/m^2^]	*K_AH_* [10^2^ m^3^/mol]	*K_BOH_* [10^7^ m^3^/mol]
control	7.11 ± 0.44	1.61 ± 0.51	3.44 ± 1.11	3.56 ± 0.81
fatal ethyl alcohol poisoning	1.16 ± 0.08	3.22 ± 0.91	0.38 ± 0.04	2.12 ± 0.26

**Table 4:** The total acid and base concentrations of the functional groups of thrombocytes and their association constants with H^+^ and OH^−^ ions.

Groups	Parameters			
	*c_A_* [10^−6^ mol/m^2^]	*c_B_* [10^−6^ mol/m^2^]	*K_AH_* [10^2^ m^3^/mol]	*K_BOH_* [10^7^ m^3^/mol]
control	3.76 ± 0.81	1.21 ± 0.23	2.88 ± 1.58	2.11 ± 0.79
fatal ethyl alcohol poisoning	5.56 ± 0.33	0.17 ± 0.03	3.47 ± 0.65	5.21 ± 0.85

## Data Availability

The data presented in this study are available on request from the corresponding author.
